# Should elephants graze or browse? The nutritional and functional consequences of dietary variation in a mixed-feeding megaherbivore

**DOI:** 10.1098/rsos.250939

**Published:** 2025-11-26

**Authors:** Hansraj Gautam, Fabio Berzaghi, M. Thanikodi, Abhirami Ravichandran, Sheshshayee M. Sreeman, Mahesh Sankaran

**Affiliations:** ^1^ National Centre for Biological Sciences, Tata Institute of Fundamental Research (TIFR), Bengaluru, Karnataka, India; ^2^ Department of Biology, University of Turku, Turku, Finland; ^3^ Sasakawa Global Ocean Institute, World Maritime University, Malmö, Sweden; ^4^ Evolutionary and Organismal Biology Unit, Jawaharlal Nehru Centre for Advanced Scientific Research, Bengaluru, Karnataka, India; ^5^ University of Agricultural Sciences, Bengaluru, Karnataka, India

**Keywords:** megaherbivore, diet, browse, nutrient, foraging, crude protein, top-down impact, elephant, grazing, carbon isotope, grazer-browser spectrum

## Abstract

Unlike specialist browsers and grazers, the diets of mixed-feeding megaherbivores are broad and complex, comprising numerous plant species of variable nutritional quality. Understanding key axes of nutritional variation in the diets of mixed-feeding megaherbivores is challenging but is crucial to understand their impacts on vegetation. Here, we revisit a long-standing debate on whether browse is more nutritious than grasses for elephants, as browse is thought to contain higher crude protein (CP). We quantified diet composition using carbon isotope analyses and analysed forage quality in 102 Asian elephant faecal samples from southern India, and found that high-browsing and low-browsing diets had similar forage quality, as indexed by nitrogen and carbon-to-nitrogen ratio. To explore the generality of this finding, we analysed nutritional differences between browse and grass across 141 plant species consumed by Asian elephants across their distribution range. We show that woody tissues and non-legume plants, which dominate elephant browse, do not have higher forage quality or CP than grasses, a trend which may be common in Asia’s mixed-feeding large herbivores. Finally, based on the observed habitat-wide variation in browsing, we provide a new framework to assess the impacts of Asian elephants on woody vegetation, with important implications for carbon cycling.

## 1. Introduction

The grazing-browsing spectrum, i.e. the variation in the consumption of grass or browse (non-grass) plants, is a key axis along which the foraging ecology of large mammalian herbivores can be differentiated [1–4]. As feeding on grass and browse involves different foraging and digestive mechanisms due to differences in their nutrition, palatability and spatial distribution [1,5,6], large herbivores are often classified into grazer, browser or mixed-feeder (grass-browse mixed consumers) guilds which also map on to their potential impacts on vegetation structure and composition [3,5,7,8]. The diets of mixed feeders are particularly interesting and challenging to study since they are complex and broad, comprising hundreds of plant species of varying nutritional quality [9,10]. This paper examines the diet of Asian elephants (*Elephas maximus*), a mixed-feeding megaherbivore, and revisits an unresolved debate on whether browse is a nutritionally better food type than grasses [10–13].

As elephants are hind-gut fermenters, their efficiency of digesting and absorbing nutrients from plant fibres is limited (unlike the ruminants that efficiently absorb nutrients in their small intestines after fermentation in the foregut [5, p. 78]). However, their fast ingesta-passage allows elephants to bulk-feed on a variety of plants for prolonged hours [9,13]; this translates into a generalist diet with massive daily food intake (approx. 140–200 kg biomass) which compensates for less efficient digestion/absorption [5]. However, all plants are not equal in nutrition [6,8,14–16], and although indiscriminate foraging maximizes biomass consumption, allocating some effort to obtaining high-quality foods helps achieve a more favourable nutrient budget and improved body condition [10,17–19].

While the availability of grasses and browse influences what elephants eat [10,20], two compilations of the composition of Asian and African savannah elephant diets indicate that modern elephants predominantly browse (based on carbon isotope signatures in teeth and bone collagen samples [11,12, p. 366]), sometimes even in grassy ecosystems [10, pp. 191–239]. Considering this, Sukumar [10, pp. 191–239, 11] asserted that browse is nutritionally more important than grasses for elephant growth as (i) it contains higher crude protein content (CP) than grasses [14], and (ii) proteins containing browse-associated carbon isotopes dominantly contribute to bone collagen in both Asian and African elephants (greater than or equal to 70% proteins came from C3 plants which are mostly browse [11,21]). Sukumar & Ramesh inferred this nutritional importance of browse after finding that browse contributed disproportionately to bone collagen despite contributing equally (as grass) to the food intake by Asian elephants in southern India ([10,11,21, pp. 203–204]; this inference accounted for the enrichment of the heavier isotope due to fractionation during metabolism [22]). Broadly, this inference appears to be in line with studies on other large herbivores finding that browse-rich diets are associated with proxies of higher forage quality (such as high CP and low carbon-to-nitrogen ratio in faecal samples), while grass-rich diets tend to be of lower forage quality ([2,23], see also [8,18,24]). However, others have cautioned against drawing such inferences about the importance of browse for elephants because elephants exhibited grass-rich diets for the majority of their recent evolutionary history [12, p. 369]—with the expansion of C4 grasslands in the Late Miocene, elephants increased C4 grass consumption till the Late Pleistocene and still retain the dental features adapted to the abrasive nature of grasses and dust [25]. Notably, Baskaran *et al.* [13], who found high percentage of grazing (85% of all scan observations) in the diets of radio-collared elephants [see also 20], contested both the importance and predominance of browse vis-à-vis grass in the diet of Asian elephants, and suggested that grass is their principal food while browsing predominates when grass is scarce [26]. We attempt to resolve this ‘browse versus grass debate’ by examining whether browsing is nutritionally better than grazing. Answering this question informs the fundamental ecology of such mixed-feeding megaherbivores [10, pp. 191–239, 13], aids in the mapping of their food resources [27] and has implications for the management of their habitats [11, p. 372, 26, pp. 13-14].

To address this debate, we examined multiple aspects of Asian elephant diets. First, we explored how elephant diet composition varied across habitats (*Question 1*). Specifically, we quantified the diet composition of wild Asian elephants from the forests of Nagarahole National Park in southern India and qualitatively compared it with previously studied grass-dominated mesic savannah habitats in the same heterogeneous Nilgiri Biosphere Reserve (NBR) landscape [13,28]. We characterized the grass-browse composition of elephant diets by analysing carbon isotopes in faecal samples. Carbon isotope analyses of faecal samples provide consistent and reliable estimates of diet composition of large herbivores [29,30]; additionally, they provide a more direct estimate of diet than analyses of bone tissues for which large adjustments are required [21, p. 538]. Next, we combined two approaches to address the browse versus grass debate. First, we analysed two proxies of forage quality, i.e. nitrogen content (N%, also a proxy of CP [6]), and carbon-to-nitrogen ratio (C : N) in the faecal samples from Nagarahole to test the assumption that browsing is nutritionally better for elephants than grazing (*Question 2a*), as suggested previously [10,11]. Faecal samples are informative of the diets of large herbivores as most of the ingesta comes out undigested, which reflects consumed forage [30–32]. Following Sukumar & Ramesh’s [11] assertion of browse being nutritionally better, we expected browse-rich faecal samples to have higher N% and lower C : N ratios, indicating better forage quality, than browse-poor samples. Second, we tested the generality of the assumption that browse has better forage quality than grasses (*Question 2b*), by comparing the nutritional quality (CP and fibre content) of grass and browse for a large compilation of plant species consumed by Asian elephants based on published literature and datasets (see §2). This analysis of food plants complements our analysis of diet quality based on faecal samples, while accounting for any biases that may arise if the quality of faecal matter is not reflective of actual forage consumed due to differences in digestibility between plant species. Finally, since dietary variation can shed light on the top-down effects on vegetation [7], we discuss how the habitat-wide variation in grazing and browsing by elephants can shape their impacts on vegetation and carbon cycling.

## 2. Material and methods

### 2.1. Study area

The study area, Nagarahole National Park (approx. 11.85–12.26° N, 76.00–76.28° E), is part of the NBR landscape, which (along with Eastern Ghats) supports the world’s largest contiguous population of Asian elephants. Dry deciduous, moist deciduous and teak plantations are the major forest types in Nagarahole, and elephants have access to both grasses and browse plants, although the forests in Nagarahole have relatively low grass abundance (fresh biomass average 202 g m^−2^, [33]). In contrast with Nagarahole forests, Baskaran *et al.* [13] observed grasses at another site in NBR to be several times more abundant (dry deciduous: 921 g m^−2^; dry thorn savannah: 524 g m^−2^) and, accordingly, observed elephants to predominantly graze. In this paper, we refer to the latter two habitats studied by Baskaran *et al.* [13] as savannahs due to their open canopy and understorey having abundant grasses, following Sankaran & Ratnam [34], whereas Nagarahole is referred to as a forest habitat. Due to grass scarcity in Nagarahole forests, we expected more browsing and less grazing in these forests compared with savannahs. To study the composition of elephant diets (grass versus browse; *Question 1*) and its association with forage quality (*Question 2*; see below), we adopted the widely used method of analysing faecal samples, which are reliably informative of diets at short-term scales (1–5 days for elephants), as majority of ingesta passes as undigested material in the faeces of large herbivores and thus reflects the consumed forage [30–32].

### 2.2. Sample collection and processing

We collected elephant dung samples from Nagarahole forests at three different time points spaced three months apart, i.e. 1 October 2022, 1 January and 1 April in 2023. These correspond to distinct seasons, i.e. namely, wet season, onset of the dry season and peak dry season, respectively. We temporally concentrated our sampling such that approximately 90% samples (91/102) were collected during a ±two week window around these dates. A majority (greater than 85%) of our samples came from the dry deciduous forests of Nagarahole. To minimize the confounding effect of age class on diet [21], we only collected dung samples with large bolus size matching that of adults. Upon finding fresh dung samples, a few hours old or from overnight, we broke open one dung bolus to collect 5–6 chunks of dung from different parts (approx. 200–300 g of fresh weight), while excluding the outer mucus layer. Samples were determined as fresh when either a defecation was observed or, in other cases, based on its warmth, smell or the shine and freshness of the outer mucus layer. We assigned an ID to each sample and noted its GPS location. Furthermore, in cases when we collected multiple dung samples which were within 50 m of each other, and thus could belong to the same foraging group, we assigned a common cluster ID to these potentially non-independent samples. Samples were air-dried in the sun to the extent possible, and then transported to the laboratory in thick-paper bags. We dried them further in a hot-air oven at approximately 60°C for approximately 60 h to remove any moisture. Next, we ground these samples using a Willey mill to obtain a fine powder for further laboratory analyses. We thoroughly mixed the contents of each sample before grinding, so that the powder obtained was representative of the collected dung. Our fieldwork adhered to research permits and India’s wildlife protection laws.

### 2.3. Quantifying grazing/browsing levels in elephant diet

To quantify the grass-browse composition of diet in Nagarahole forests (*Question 1*), we performed mass spectrometry analyses of faecal samples to obtain δ^13^C, i.e. the ratio of the heavy ^13^C and light ^12^C stable carbon isotopes. We quantified the δ^13^C for faecal samples with respect to the reference standard (Vienna Pee Dee Belemnite) as


$${\text{δ}}^{13}\text{C}=[({(}^{13}\text{C}{/}^{12}\text{C) sample)}/({(}^{13}\text{C}{/}^{12}\text{C})\text{standard})-1]\times 1000.$$

Here, ^13^C and ^12^C represent masses of the two carbon isotopes measured in the sample and the standard.

C3 plants are more depleted in the heavier isotope and thus yield lower (more negative) δ^13^C values. For instance, C3 browse plants in this region have average δ^13^C value of –27.2‰ while C4 grasses have average δ^13^C = −12.8‰ [22]. When a mixture of C3 and C4 plants (e.g. mixed diet) is analysed, a range of intermediate values of δ^13^C values can be obtained, depending on which type of plants dominate the plant mixture. Controlled feeding experiments on ungulates show very minor differences in δ^13^C between actual forage consumed and faeces, regardless of whether the given forage was dominated by C3 or C4 (consistent difference of approx. −0.9‰ in [30] and –0.8‰ in [29]). Thus, the δ^13^C analysis of faeces reliably quantifies diet composition. Here, we qualitatively inferred approximately 50% contribution of C4 grasses and browse to elephant diet when δ^13^C was approximately −20, following Codron *et al.* [35], although exact composition should ideally be modelled from mixtures of local vegetation.

### 2.4. Quantifying nutrients in diet

To study the nutritional quality of diet (*Question 2a*), we examined two metrics in faecal samples: nitrogen content (N%) and carbon-to-nitrogen ratio (C : N), which are positive and negative correlates of diet quality, respectively [31,35]. We quantified the faecal N% and C% (to obtain C : N ratio) using a Leco TrueSpec CN analyser, using EDTA LCRM^®^ as the reference standard.

### 2.5. Processing data on the nutritional quality of grass and browse plants eaten by elephants

While several studies highlight the efficacy of faecal analyses in understanding the composition and quality of the diets of large herbivores [30–32], the excreted plant material in faecal samples may not fully reflect the quality of consumed forage (and potentially represent a lower-quality fraction of the forage consumed) due to differences in digestibility between plant species or parts. Therefore, we complemented the above faecal analyses by analysing a large compilation of the nutritional values of browse and grass species reported to be consumed by Asian elephants. These nutritional values (CP and fibre content) were from different habitats (see below) and thus help in testing the generality of the assumption that browse is more nutritious than grasses (*Question 2b*). This compilation of nutritional values of elephant food plants was obtained from two existing databases collated using data reported in previous studies: (i) MegaFeed, a database of food plants of extant Proboscidea species, from which we extracted the list of recorded food plants of Asian elephants [36]; and (ii) PNuts, a global database of plant nutritional values for different type of plants and plant parts or organs [37]. PNuts has been used in a previous study on nutritional values of forest elephant foods [38]. PNuts provided values of CP, acid detergent fibre (ADF) and neutral detergent fibre (NDF) for plant species consumed by Asian elephants as indicated in MegaFeed. We have provided the complete list of plant species and nutritional values of each subcategory in the electronic supplementary material, List S1, tables S3 and S4. CP in this database was either the reported CP or, in cases when original studies only reported N, calculated using a conversion factor of 6.25 following the Kjeldhal procedure [16,39]. Plant species names were updated and homogenized following the taxonomy in World Flora Online (https://wfoplantlist.org/). ‘Liana’, ‘Tree’, ‘Shrub’, ‘Legume’ and ‘Herb’ in the ‘Plant_type’ column in electronic supplementary material [40] were classified as ‘browse’. Some plants consumed by elephants can also be found outside the distribution range of elephants, either because they are species with rather large distributions or because they are species that have been introduced. Because plant nutritional values are determined not just by plant type but also by their growing environmental conditions [16,37], we analysed our data in two ways. We first analysed the full ‘global dataset’ that also contained samples from outside the range of Asian elephants. However, since these samples could potentially make the estimates unrepresentative of the range of Asian elephants, we also analysed samples from only Asia, called ‘Asia-only’ dataset.

Fibres were analysed in addition to protein because low values of ADF and NDF indicate higher digestibility of forage [16], thus higher assimilation of protein and other nutrients. We first explored nutritional differences between (i) grasses and browse. We then explored different subcategories of browse for comparison of (ii) grass with non-legume browse and legume browse, since legumes are rare in the mesic habitats of Asian elephants, and comparison of (iii) grass with subcategories of browse plant parts (leafy browse and woody browse that included bark, branch, stem, etc.) since a lot of browse eaten by elephants includes woody tissues [41,42] which may be low-quality foods. Fruits and seeds were excluded from our analyses due to small sample sizes, but their values are reported in electronic supplementary material, tables S2 and S3.

### 2.6. Statistical analyses

First, to test if diet quality within each season was associated with diet composition (in terms of browse versus graze, *Question 2a*), we separately modelled N% and C : N in elephant faecal samples using linear mixed models (LMMs, lmer function in lmerTest package [43]), by including δ^13^C, season and their interaction as fixed-effects predictors and sample cluster as random effect. The δ^13^C × season interaction would allow the relationship (slope) between δ^13^C and diet quality to vary in different seasons. In these two LMMs, we modelled the intercept term by limiting δ^13^C (continuous predictor) to its smallest negative value in the dataset (rather than 0 which is an unrealistic δ^13^C value for these plants). To infer the significance of effects, we obtained ANOVA tables using the *anova* function with Type III SS appropriate for unbalanced designs (obtained using Wald distribution). We inferred the effect size or slope from the estimates of fixed effects in the LMMs. We log-transformed C : N values to reduce heteroscedasticity and skew in the distribution of residuals in these LMMs. Following previous studies using carbon isotope analyses to study elephant diets [11,21], we limit our inferences regarding grazing to only C4 grass consumption. Second, we analysed data from the two global databases mentioned above to further examine any generalizable nutritional differences between browse and grass. We analysed plant nutritional values (CP, ADF and NDF) in Asian elephant food plants, along the three categorizations mentioned above. We used pairwise *t*-tests in R to test statistical differences between the means of different categories: (i) grass and browse; (ii) grass, legume browse and non-legume browse; and (iii) grass, leafy browse, woody browse. We did these analyses with plant nutrient data coming from two spatial extents: Asia only and global. Data and R scripts used in this paper are available in [40].

## 3. Results

### 3.1. Grazing and browsing composition of elephant diet in forests and savannahs

**Figure 1.**
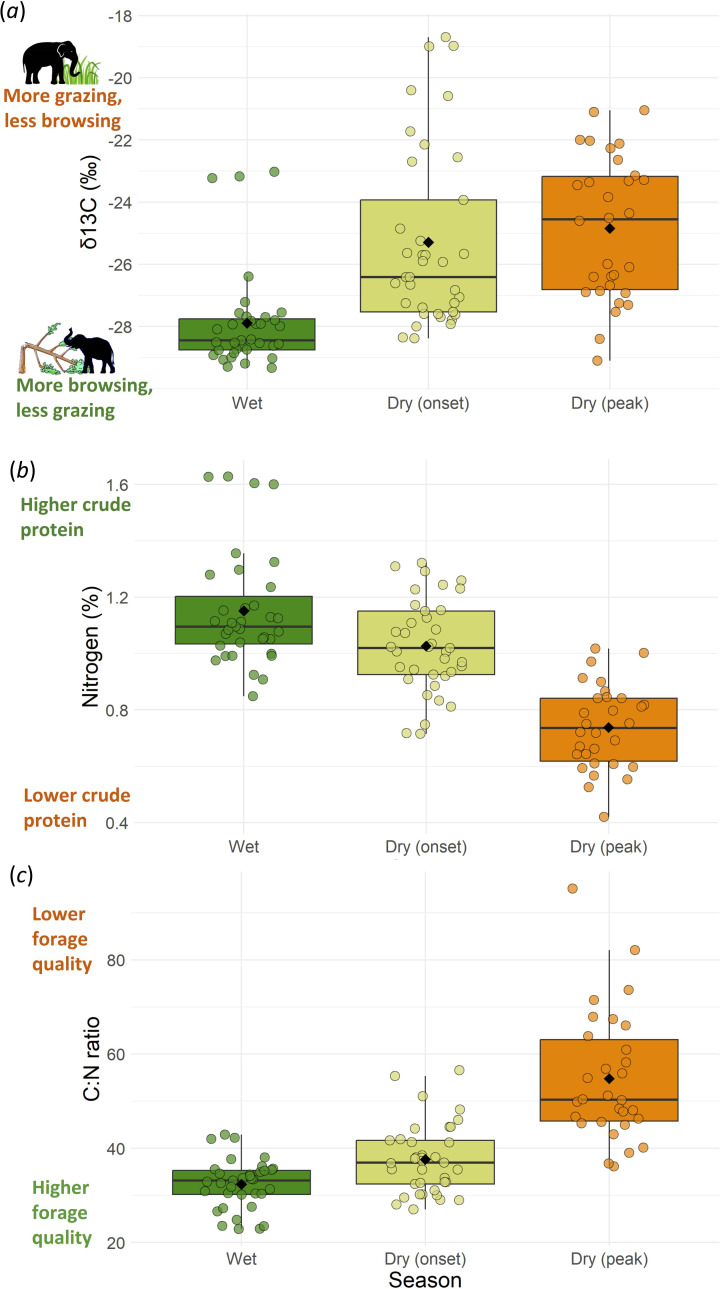
Seasonal variation in grass-browse composition and forage quality of elephant diet inferred from faecal samples from wet, early dry and peak dry seasons. As the dry season progresses (*a*) consumption of C4 grasses increases, (*b*) nitrogen content declines and (*c*) C : N ratio increases. Each circle represents a faecal sample, while black diamonds represent seasonal means and the horizontal line represents the median.

In our quantification of the composition of elephant diet in Nagarahole forests (*Question 1*), we found that the overall mean δ^13^C of elephant dung was −26.05‰, indicating that elephants had a heavily browse-dominated diet (greater than or equal to 75% browse). Such a browse-rich diet in these forests contrasts with the grazing-dominated diet observed in the nearby savannahs of the same NBR landscape (δ^13^C approx. −21‰ to −14‰, i.e. approx. 50% to greater than or equal to 90% grazing in [28], greater than 85% grazing in [13], electronic supplementary material, text Sa). Elephant diet also varied seasonally and both the proportion of browsing and the forage quality of elephant diet declined from wet to dry season (figure 1, electronic supplementary material, text Sb).

### 3.2. Browse-heavy diets do not have higher crude protein content

**Figure 2.**
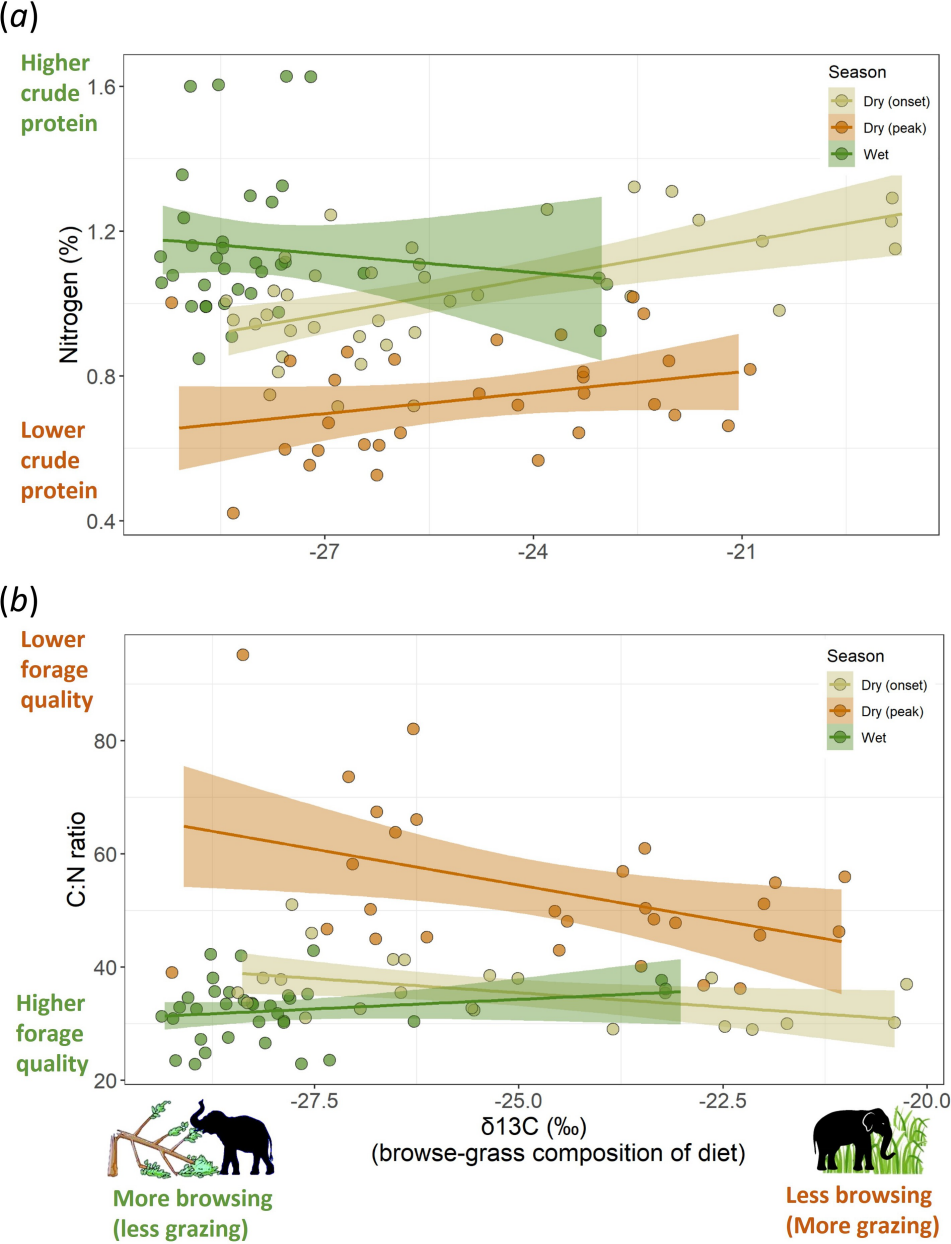
The relationship between grass-browse composition of elephant diet and its nutritional indicators, (*a*) N% and (*b*) C : N ratio. Each circle represents a faecal sample.

To evaluate whether browsing provided better forage quality than grazing (*Question 2a*), we analysed within-season association between diet composition and quality estimated from the faecal samples. In our examination of whether diet composition varied with two proxies of forage quality, N% and C : N ratio, we found that forage quality did not increase with browsing. N%, also a positive correlate of CP, was not higher in samples showing browse-heavy diets (i.e. more negative δ^13^C), as evident from the absence of a negative relation between δ^13^C and N% (electronic supplementary material, tables S1a and S2a, figure 2*a*). Instead, a weak positive relation suggests that browse-rich diets were of rather lower forage quality (estimates for δ^13^C’s effect on N% = 0.03). Declining browsing levels (i.e. increasing δ^13^C) were associated with lower forage N% in wet season but increased N% in dry seasons, although this interaction effect was not significant (*p* = 0.07, electronic supplementary materials, tables S1a and S2). C : N ratio, a negative correlate of forage quality, declined weakly when browsing decreased, indicating that lower-browsing diets were of marginally higher quality (estimate for δ^13^C effect on C : N = −0.04). This relationship varied seasonally (electronic supplementary material, table S1b), with a decline in browsing levels leading to a weak increase in C : N (i.e. reduced forage quality) during the wet season but reducing the C : N in the dry season (figure 2, electronic supplementary material, table S2b). Overall, the small effect sizes for the effect of δ^13^C on these proxies of CP and forage quality imply that the variation in forage quality was poorly explained by grazing/browsing levels (electronic supplementary material, table S2). Put simply, higher browsing did not provide more CP or better forage quality.

### 3.3. Forage quality of grass and browse food plants

**Figure 3.**
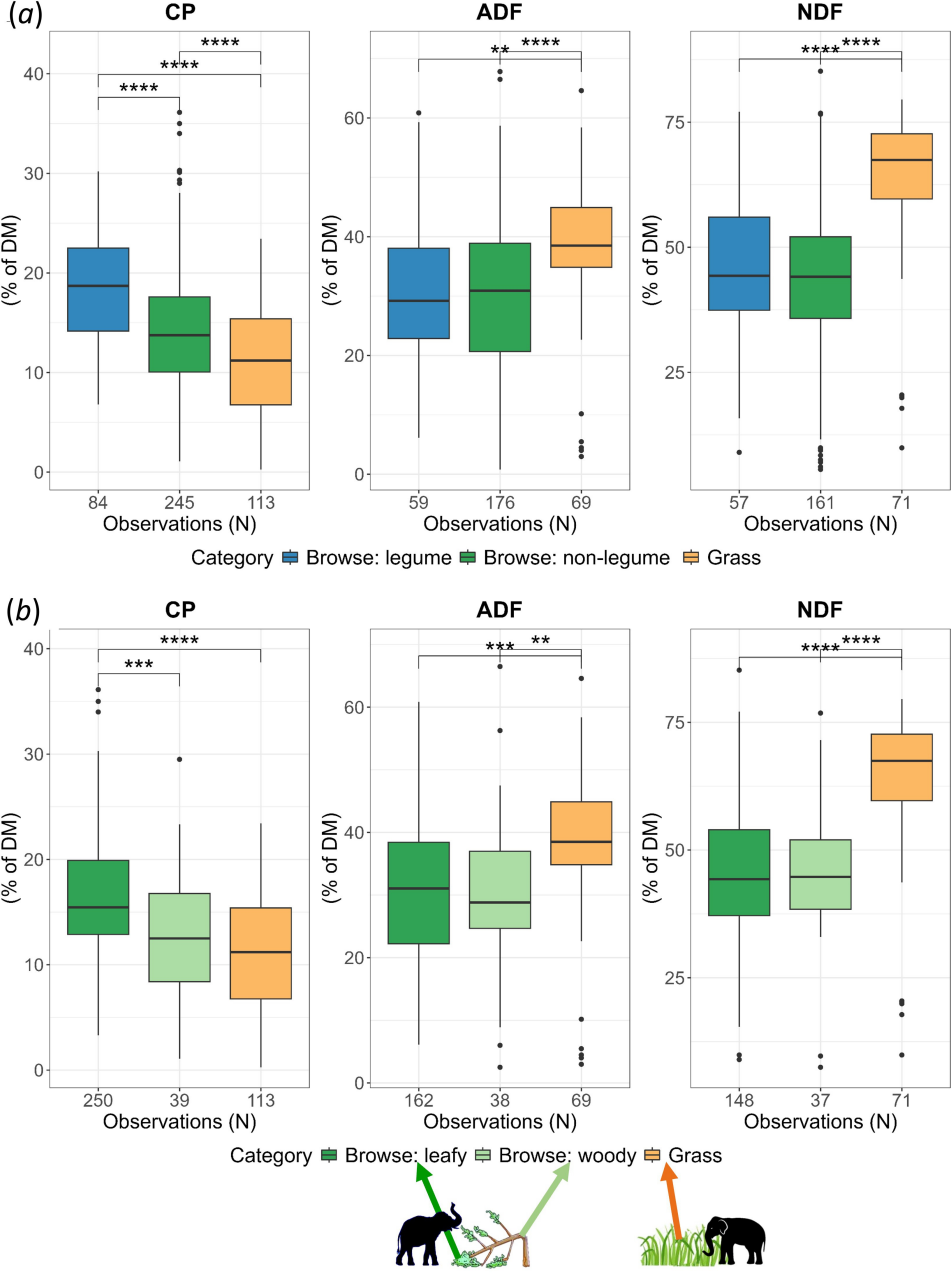
Nutritional values of elephant food species from samples at global extent. (*a*) Legume and non-legume browse have higher CP and lower fibre content than grass; (*b*) leafy browse has greater CP and lower fibre content than grass while woody browse has similar CP as grasses. *p*-values calculated using the *t*-test indicate statistical significance between the mean of the two groups. Significance levels: .*p* < 0.10; *p < 0.05; **p < 0.01; ***p < 0.001; ****p < 0.0001.

In our broader analysis of the forage quality of plants consumed by Asian elephants (*Question 2b*), we found that browse had higher CP and lower fibre content in both Asia-limited and global samples (electronic supplementary material, figure S1; mean, s.d. and *n* presented in electronic supplementary material, tables S3 and S4). However, as we expected browse to be a nutritionally heterogeneous category, we further explored nutritional differences along two sub-categories of browse: legume versus non-legume and woody browse versus leafy browse. Legumes clearly had higher CP (18.5% of dry mass) and lower fibre than grass as well as non-legume browse, whereas the CP in non-legume browse (14.3% of dry mass) was only marginally higher than grass (11.6% of dry mass) in the full global dataset (figure 3*a*, electronic supplementary material, table S4; similar trends in Asia-limited data: electronic supplementary materials, figure S2 and table S3). While leafy browse had higher CP than grass, woody browse, which dominates elephant browse (§4), did not have significantly higher CP, although it had lower fibre content than grasses (full global data: figure 3b, electronic supplementary material, table S4; similar trends in Asia-limited data: electronic supplementary materials, figure S2 and table S3). Thus, woody tissues and non-legume plants, which often dominate the browse component of Asian elephant diet (§4), do not provide appreciably higher CP than grasses.

## 4. Discussion and conclusion

Here, we quantified the contribution of the grass and browse components to the diet of Asian elephants in a forest habitat (*Question 1*) and revisited the debate on whether browse food plants provide greater protein content and are thus nutritionally more important than grasses for elephants (*Question 2* [10,11,13]). We examined two proximate lines of evidence: (i) the association between carbon isotope ratios (proxies of grazing/browsing levels) and the nutrients in faecal samples indicative of elephant diet (*Question 2a*); and (ii) comparison of the forage quality of browse and grass species consumed by Asian elephants (*Question 2b*). We found that although elephant diets in these forests were dominated by browse, browse-heavy diets (as inferred from lower δ^13^C) did not have appreciably higher CP (inferred from N%) and forage quality (inferred from both high N% and low C : N), which contradicts the presumed nutritional advantage of browse. This trend appears to be generalizable since our analysis of nutritional values of elephant food plants showed that CP content in browse, especially woody browse, was not necessarily better than grasses. We also found that browsing in elephant diets in Nagarahole forests was more common than in the savannahs of the NBR landscape (*Question 1*). Such dietary variation has implications for the impact of these megaherbivores on vegetation.

### 4.1. Browsing is not nutritionally better than grazing

The similarity in the CP content and forage quality of browse-rich and browse-poor diets (figure 2) contradicts the presumption that browsing has higher nutritional and CP value than grazing [10,11]. Even in the peak dry season, for which the nutritional advantage of browse was especially emphasized earlier [10,11], browse-rich diets did not correspond with higher forage quality in our study. This contradiction is not surprising since elephant browse is a nutritionally heterogeneous category that also contains many low-quality food items. It is worth noting that Sukumar’s inference [10] of higher CP in browse was based only on leaves (appendix 1 in [14]) which often represents only a small subset of the elephant diet, especially in the dry season. Woody food items like stems, twigs and bark can constitute as much as leaves, or even dominate the browsing component of elephant diets (e.g. 65% of all browse in moist and 37% in dry forests was woody browse in [41, p. 96], 66% in [42, p. 54], 50–94% in [19]), but may have poor CP content and forage quality. Indeed, in our results on nutrient value of elephant food plants, browse was a nutritionally heterogeneous category, with woody browse having similar CP as grasses and only leafy browse having higher CP (figure 3). Similarly lower CP value of woody browse vis-à-vis leafy browse is also seen in the diet of Africa’s black rhinoceros [15]. CP in leafy browse seems to be higher than in ‘fruit and seed’, although fibre contents suggest that the latter was more digestible (electronic supplementary materials, tables S3 and S4). These findings suggest a large nutritional asymmetry along the plant-part axis vis-à-vis the grass-browse axis, which could influence feeding preferences for browse or grass. Furthermore, while elephant browse as a category did have higher CP (electronic supplementary material, figure S1, possibly because our dataset was dominated by leafy browse, see figure 3), this advantage declined sharply when we excluded legumes (figure 3, see also [16, p. 645]), which are usually rare in mesic habitats of Asian elephants (in NBR, less than 3% tree saplings in Nagarahole are legumes, based on data in [44]; less than 10% trees in Mudumalai National Park are legumes out of which less than 2% are nitrogen-fixers [45]; less than 2% legumes were reported in elephant browse in Wayanad Wildlife Sanctuary [42]; and legumes are also rare in Corbett National Park, North India [46, p. 75], see also [47]). Thus, at least in terms of N% and C : N, most of the browse eaten by these bulk-feeding mixed feeders does not provide greater nutritional value than grasses (but see [8] for browser versus grazer guilds). Therefore, we think that such similarity in the nutrient value of grazing and browsing may be common in other large mammalian herbivores in Asia, many of which are mixed feeders [48]. This likely scenario is supported by a previously reported similarity in the CP of grass (15%) and browse (14–15%) plants consumed by a range of herbivores from across the world [16]. While lower fibre content may appear to make browse more digestible than grass, it must be noted that we did not examine defensive or structural compounds such as tannins and lignins. These compounds often occur at higher concentration in browse [16] and can reduce the digestibility and protein intake from browse [6,18], thus potentially cancelling out any marginal advantage due to lower fibre or higher CP content. Considering such effects of defensive compounds, Codron *et al.* [6] suggest that the fibre in grasses may be more digestible, making their energy yield comparable or even higher than browse.

Thus, we are faced with two contradictory patterns in the browse versus grass debate: (i) browse-rich diets are not associated with greater CP content or forage quality than browse-poor diets (figures 2 and 3b), but (ii) browse-associated proteins contribute dominantly to elephant bone collagen [11,21]. Contesting Sukumar’s inference based on δ^13^C analyses of bone collagen, Baskaran *et al.* [13] invoked the lack of knowledge of the ranging history of the sampled dead elephants as browse-dominated signatures could arise if elephants used browse-rich habitats. While we concur that ranging history is informative, we note that this browse-dominated signature is very common in collagen of African savannah and Asian elephants [10,12]. Future studies can reconcile these two contradictory patterns by exploring: (i) if carbon from grass and browse gets differentially allocated in body tissues other than collagen; and (ii) if grasses provide higher instant energy/resources which are utilized immediately and not allocated to body tissues. Future studies can also add insights by replicating our study in other mixed feeders, and advancing it by using methods that allow more detailed taxonomic characterization of diet (e.g. DNA-metabarcoding [4,49]). Simultaneously collecting faecal and plant samples from multiple sites and seasons is desirable, as our global dataset does not control for spatio-temporal variation in CP. Finally, the negative effects of secondary metabolites on forage quality also need to be considered since browse has condensed tannins and lignins that reduce digestibility and food preference [2,6,16].

### 4.2. Functional implications of the dietary variation across forests and savannahs

**Figure 4.**
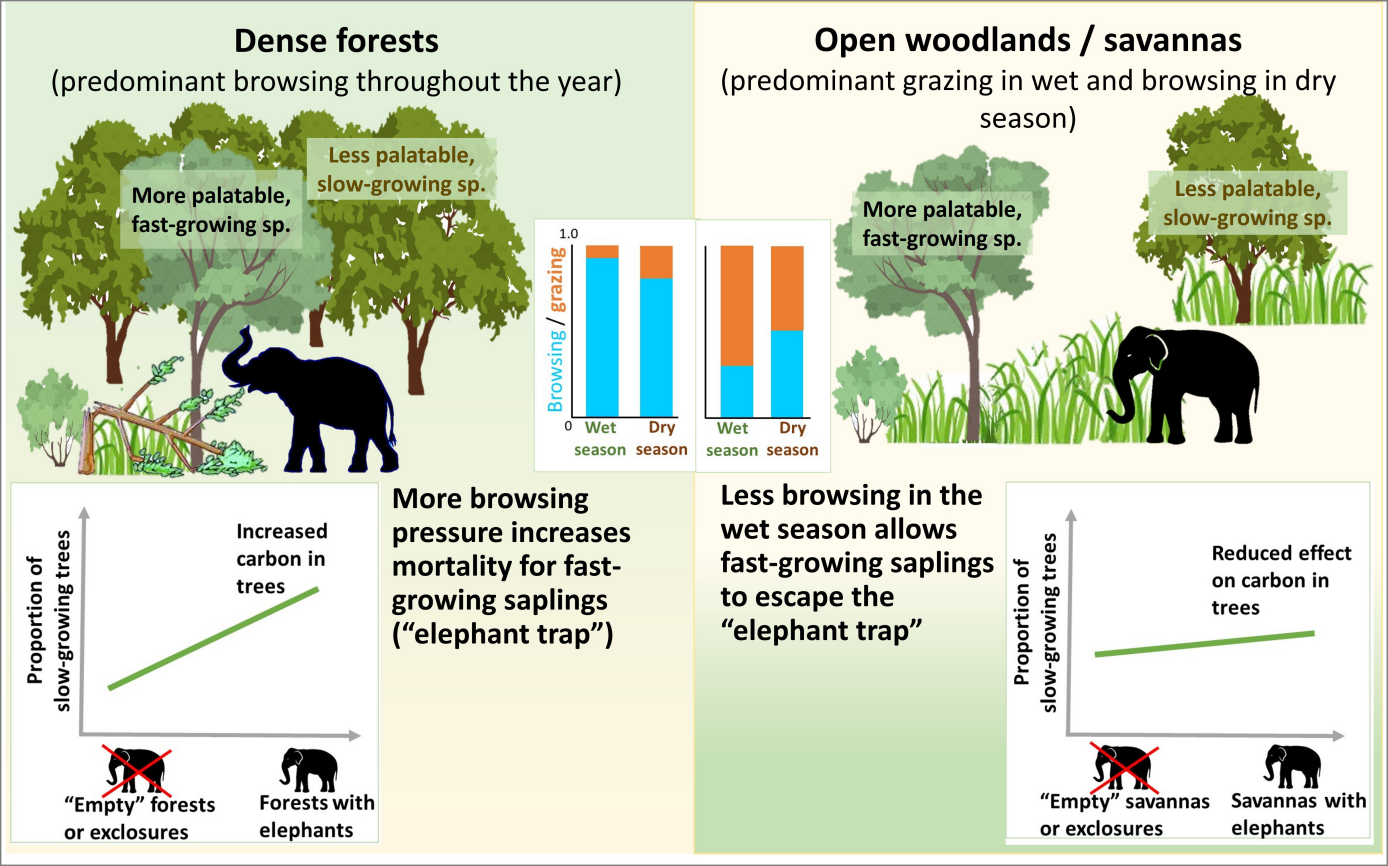
Expected impact of mixed-feeding megaherbivores on woody species composition and above-ground carbon in grass-scarce forests versus grass-abundant savannahs. Left: the preferential browsing on fast-growing species with low wood density would increase carbon-dense tree species as compared with the elephant ‘empty forests’ or exclosures. Right: more grazing would weaken the browsing effect of elephants on tree carbon as fast-growing, low-carbon trees escape the browse trap. The grazing/browsing levels shown in the inset are qualitative representations based on findings from NBR.

The more predominant browsing observed in the grass-scarce Nagarahole forests than in the grass-abundant savannahs of the NBR landscape suggests that browsing declines with increasing grass abundance (findings from NBR reviewed in electronic supplementary material, text Sa, see also [50, p. 10]) for grazing patterns in Uda Walawe, Sri Lanka). Another contrast between these savannahs and forests is the seasonality in grazing/browsing levels: grazing in savannahs dominates elephant diet in the wet season but declines in dry season [13,21], whereas elephants in Nagarahole forests have a persistently browse-dominated diet throughout the year (figure 1, eletronic supplementary material, text Sb; all-year predominant browsing also reported from Thailand’s forests [51]). Such habitat-dependent dietary variation can shape the impact of such mixed-feeding megaherbivores on woody vegetation (figure 4).

In figure 4, we present a new framework to assess the impacts of such mixed feeders on woody vegetation and carbon in the savannahs and forests of Asia, based on the dietary variation across habitats. In dense forests like Nagarahole, the persistently predominant browsing (due to grass scarcity) creates a mortality filter or an ‘elephant trap’ for saplings/recruits [52,53]. Recently, Berzaghi *et al.* [38] showed that African forest elephants seeking high-quality browse cause greater damage to trees of fast-growing species as they are more palatable (due to lower levels of fibres and chemical defences). Such preferential browsing enhances above-ground carbon storage by shifting tree communities towards slow-growing trees (with higher wood density and carbon storage than faster-growing species) which are less preferred due to higher levels of fibre and defences [38]. Given that Asian elephants are browsers throughout the year in forests, they also may potentially promote slow-growing species through the year-round operation of this browsing trap (figure 4, left). Some empirical support for such impact comes from preferential browsing causing more damage to tree saplings in early successional forests (dominated by fast-growing species) than in mature forests in Malaysia [53]. In contrast, in savannahs, some fast-growing tree recruits may escape this mortality filter in the productive season when elephants primarily graze. Such seasonal relaxation of tree mortality may limit the ability of elephants to shift woody community composition towards slow-growing species in Asia’s savannahs (vis-à-vis forests). In other words, their role in such above-ground carbon sequestration is expected to be lower in mesic savannahs than forests (flatter slope in figure 4, right). Secondly, such tree mortality may be critical for the emergence of canopy gaps in forests inhabited by elephants, thus enhancing plant diversity by promoting favourable sunlight niches for shade-intolerant species [52]. Thirdly, these destructive foragers leave behind trails of high disturbance [9], thus promoting heterogeneous microclimates for a diverse understorey, through woody mortality in forests and grass removal in savannahs. Intensive browsing by elephants could also influence the structure of woody plants, for example, tree height, height-to-girth ratio and crown shape [54,55]. Such diet-mediated top-down impacts of mixed feeders on vegetation can be tested through observations of foraging [53], exclosure experiments or dynamic vegetation models [56], and could refine the recent emphasis on the global role of such megaherbivores in shaping vegetation and carbon dynamics.

### 4.3. Conclusion

Our results clarifying the fundamental ecology of this mixed feeder have implications for habitat management. In light of the similar nutritional value of elephant browse and grass, we suggest a moderation in the emphasis on either browse [11, p. 372] or grass [26, pp. 13–14] abundance for the management of Asian elephant habitats. The question of which habitats or mosaics provide nutritionally better forage—and thus can better support elephants—is difficult to address assuming either grass or browse abundance as the limiting factor, especially because elephant diet varies widely with vegetation composition. Replicating our study in multiple sites, and extending it to more nutrition variables like minerals [57] and secondary metabolites, can be a useful approach to assess forage quality, including in other mixed-feeding large herbivores of Asia.

## Data Availability

Datasets used are available online and cited in the main text. Supplementary material is available online [58].
